# Treatment of Dental Caries, When the Cavity Extends Nearly to the Nervous Pulp

**Published:** 1849-07

**Authors:** C. O. Cone

**Affiliations:** Baltimore.


### ARTICLE V.

*Treatment of Dental Caries, when the Cavity extends nearly to the Nervous Pulp.* By C. O. CONE, M. D. of Baltimore.

THE dental, or nervous pulp of a tooth, in common with all vascular tissues, is liable to inflammation, modified, however, in character and result, not only by its anatomical position, but by the nature and cause of the inflammation, as well as by the constitutional habit of the patient, and the pathological condition in which the pulp is found at the period when the cause becomes an irritant.

Although the irritation, and eventual inflammation of the nervous pulp of a tooth, produced by caries, may

30*

have the same final termination as might arise from a metallic plug, namely—suppuration of this tissue, and destruction of the vitality of the crown of the tooth; yet, the two differ not less in themselves, than in their pathological history and treatment.

The anatomical and pathological condition of the parts, in the one case and in the other, are very different, and hence, the greater liability of immediate general inflammation in the one than in the other. When caries has penetrated almost to the nervous pulp, or, even through the dentine to the pulp cavity, local inflammation more frequently supervenes, than would be the case if the same amount of irritation was communicated by a metallic plug. If the decay does not penetrate the pulp cavity, local and ossific inflammation is the result, but, should the decay advance rapidly, and even penetrate the pulp cavity, local inflammation is always the result for a time of more or less duration, according to circumstances. The decay has formed a passage for the escape of any discharge poured out from the inflamed surface of the irritated part, as well as a vacuum for the increased dimension of the nervous pulp from augmentation in the size of the capillary vessels of the same; and the inflammation is, by this provision, confined for a time to the pulp situated near the opening.

When a plug is demanded near the nervous pulp, this tissue is rendered more susceptible to morbid influence from this source, not only from the preparation of the cavity, preparatory to plugging, but the disease which made the demand for the operation, has increased the susceptibility of the organ to take on an inflammatory action. The plug is introduced, and the prolonged irritation to which the nervous pulp is subjected from the loss of dental substance, and the superiority of metal as a conducting medium, institutes at first slight inflammation in the nervous pulp: and the bony wall in which it is enclosed, being entire, any increase of size although local at first, or any discharge from one part, finding no escape, presses equally on all parts: thus the inflammation becomes general, and the whole organ involved in disease, by operations, the design of which was their preservation—this is well shown in the following case:

During the month of March, 1843, Mr. S. of Mass.—about twenty-three years of age, sanguino-nervous temperament, with a denture full and regularly arranged, and holding a position between the first and second class as respects their physical characteristics—requested me to examine the right first superior molar, which had recently admonished him of the presence of disease.

The tooth was marked by a broad decay on its labial surface, extending nearly to the neck of the same In removing the decayed bone, preparatory to plugging, considerable pain was experienced, but no indication of an exposed nerve was manifest. The cavity was filled with gold and the patient dismissed.

The next day the patient returned with a request that I should again examine the tooth for another decay; stating the grounds of his suspicions to be sharp paroxysms of pain, which were felt on receiving cold or warm aliments, or when the tooth underwent any change of temperature. I assured him that no other decay existed in that tooth, and endeavored to explain to his mind, that the pain he experienced, was from the loss of tooth substance, and the superior readiness with which the metal employed transmitted changes of temperature over the sound portions of the tooth. With this explanation, and an expectation that the annoyance would gradually subside, he left.

After two days had elapsed, he again returned, complaining that the pain had increased both in duration and intensity. Local depletion, and sedatives were prescribed. The pain continued, and the plug was removed, but the inflammation had become general, and the pain constant and throbbing, with a slight elongation of the tooth from the peridental membrane becoming inflamed. The tooth was extracted and examined.

The peridental membrane showed marks of inflammation. On breaking open the tooth, a thin plate of bone separated the plug from the nervous pulp. This tissue was red, and the capillary vessels increased in size, but no pus could be detected with the eye.

Great difference exists in the susceptibility to the morbid effects of a metallic plug when introduced in close proximity with the nervous pulp, varying with different classes of teeth, with different temperaments of individuals, and still more with the pathological condition of the organ at the time when the plug is introduced.

Patients whose temperaments approach the nervous, and whose teeth are highly organized, are much more likely to suffer, from a close approximation of a plug to the nervous pulp of a tooth, than others whose temperaments are less irritable, and whose teeth are more dense, and the mineral ingredients better supplied.

When a tooth is attacked by caries, three vital actions are evinced: first, sensibility is increased; secondly, irritation is transmitted to the pulp; and, thirdly, this impression induces the pulp to renew its formative function, and thereby recede from the source of its annoyance, behind a plate of secondary dentine, formed from its substance. It is upon the extent to which the third division, or protective process has advanced, and the vascularity of the same, together with the absence, or increase of sensibility of the organ from the extent and morbid influence of the decay, that the extent to which a tooth is made to suffer pain from the introduction of a metallic plug in a deep-seated caries depends.

Irritation to the nervous pulp of a tooth, from the loss of dentine being supplied by a metallic body, terminates in one of three ways, namely:

First, if the plug be small, the nervous pulp becomes inured to this source of irritation; but if the metallic body is large and near the nervous pulp, and the progress of the decay such as to convert this tissue into secondary dentine, its density may be greatly increased, and its vitality, immediately beneath the plug and over the nervous pulp, lessened: thus the delicate tissue is protected from the irritating influence of the metal, as illustrated by the following case:

Mrs. M., of Mass., about twenty-five years of age, of a nervous-bilious temperament, and of general good health, with teeth of the second class, consulted me in relation to a decay on the anterior approximal surface of the left second inferior molar. The tooth had been plugged some three years previously, on its grinding surface, by a dentist of high eminence in Boston. The plug was very large, firmly packed, and the operation in all its relations well executed. The crown of the tooth was marked in several places by light yellow and opaque spots, indicating, at those points, the destruction of the nutritive vessels.

In preparing the cavity in the anterior approximal surface of the tooth, it was deemed best to unite the cavity on the grinding surface with the one which was being prepared, making of the two cavities a single excavation. In carrying this design into execution, the plug in the grinding surface of the crown was removed. The perpendicular walls of the cavity in the grinding surface of the tooth were sound, and free from any marks of decay, except where the disease had penetrated from the anterior approximal surface. The floor of the cavity was discolored, presenting a light purple complexion, and on applying an instrument to its centre, it glided from its surface as from a vitreous body, until it nearly reached the circumference of the cavity, where the complexion of the dentine faded, and became less dense but more sensitive.

I learned from the patient, that after the plug which I had just removed was first inserted, the tooth gave her considerable annoyance for some time, which at one period almost determined her to submit to its extraction, but that, at length, it gradually ceased to give her pain.

The explanation of this case is plain. The decay in the tooth had produced irritation, which had stimulated the nervous pulp to renew its formative function, and thus a portion of this tissue was converted into secondary dentine previously to the introduction of the plug. The superiority of the metal as a conducting medium, instituted some inflammation, and the vascular canals which mark this dentine, became deeply injected, and afterwards calcified, thus increasing the density of the intervening osseous plate.

Secondly, the pulp may become calcified or converted into dentine, *after* the plug has been introduced, thus placing between it and the metal an osseous partition, sufficiently dense and thick, behind which to retire from the irritating presence of the foreign body. We have instances of this termination following irritation conveyed to the nervous pulp, when a portion of the dentine of a tooth has been lost by abrasion, or wearing of the teeth by mastication, or even after the use of the file. Indeed, almost every tooth that is extracted from the effects of caries, if the nervous pulp be examined, will be found more or less calcified opposite the decay.

Thirdly, if the irritation of a metallic plug be severe and active, or, even if the irritation be slight at first, yet from a faulty condition of the secretive vessels, or from irritability of the organ, dependent on the morbid effects of decay, or from constitutional tendency, it be long continued, it may terminate in suppurative inflammation. To prevent this last result following an operation, it becomes the duty of the dentist to seek for and employ such means as shall best secure the patient against such a result, when the case admonishes him to adopt a precautionary course.

When a carious tooth has been examined, and the decay has approached so near the nervous pulp as to exhibit secondary dentine, or the irritation of decay has rendered the tooth morbidly sensitive to changes of temperature, and an increased susceptibility to inflammatory action, some good non-conducting substance should be placed between the thin plate of bone covering the nervous pulp and the metallic plug, that the irritation dependent on the presence of the metal may be lessened.

Filamentous asbestos was introduced to the notice of the profession in 1840, by Dr. Solyman Brown, as a proper agent, from its well known qualities, to be employed in such cases.* Dr. L. Adams, of this city, has employed successfully, for some time, a layer of *very fine* textured cork. In 1848, the Drs. Nelson, of Albany, informed me by letter, that for some time they had employed, for the purpose now under discussion, a layer of oiled silk, with highly satisfactory results. Dr. J. H. Foster, (I think it was,) stated before the American Society of Dental Surgeons, that he had employed a thin layer of "Hill's stoppings" for this purpose, in one or more cases. Dr. C. A. Harris proposes the employment of *gutta percha*, in the form of a saturated solution, prepared with chloroform.

Each of these agents, possesses, under certain circumstances, peculiar advantages; but some are more exposed to practical objections than others, from their destructibility and want of pliability.

If the agent used cannot be pressed in all directions, so as take the shape of the floor of the cavity which it protects, it attains only one end, corrects only one evil, and leaves another liable to occur at such parts of the cavity as the material may not come in contact with. I now refer to the possibility of a tooth decaying after it has been plugged in such a manner *as to exclude all external agents*—a possibility which I believe *may exist* where a *vacuum* is formed between the plug and the walls of the cavity, *in certain conditions of a tooth.* Moreover the bulk of material demanded, when some of these agents are used, precludes their employment in the anterior teeth, and even in the posterior teeth except in large cavities.

For the above reasons I have recently been employ-

* American Journal of Dental Science, vol. i, p. 241.

ing, with the most gratifying success in all cases where this precautionary practice was apparent, oil-silk in layers differing in thickness, according to the peculiarity and condition of the case; but that "Hill's stoppings," and preparations of *gutta percha,* may not be used with equal advantage and success, my practice and experience will not admit of my expressing an opinion. That these or similar agents, recommend themselves to the dental practitioner to be judiciously employed, in both relieving the sufferings of patients, dependent on dental operations, and in enhancing the success of the latter can no longer be a subject of debate in practice.

